# Henoch-Schönlein Purpura in an Obese Adult with Elevated Transaminase: A Case Report and Literature Review

**DOI:** 10.4314/ejhs.v32i4.25

**Published:** 2022-07

**Authors:** Andrew Limavady, Ketut Suryana

**Affiliations:** 1 Medical Doctor, Puri Raharja General Hospital, Denpasar, Indonesia; 2 Division of Allergy and Immunology, Department of Internal Medicine, Puri Raharja General Hospital, Denpasar, Indonesia

**Keywords:** Henoch-Schönlein Purpura, adult, elevated transaminase, obesity, case report

## Abstract

**Background:**

Henoch-Schönlein Purpura (HSP) cases are uncommon among adults due to their self-limiting nature and difficulty in diagnosis. The involvement of the hepatobiliary system and the effect of obesity is still not well understood among adults.

**Case Description:**

A 28-year-old obese male presented with an acute onset of generalized palpable purpura, fever, abdominal discomfort, and arthralgias. Laboratory examination indicated leukocytosis (43,100/µL), thrombocytosis (550,000/µL), and elevated transaminase levels (AST 125 U/L, ALT 131 U/L). Skin biopsy revealed perivascular inflammatory infiltrates in the superficial dermal vessels. Treatment of corticosteroids and antibiotics resulted in progressive clinical improvement.

**Conclusion:**

Although rare in adults, recognizing multiple presentations of HSP allows early diagnosis. This case highlights elevated transaminase levels and obesity contributed to increased inflammatory responses that may complicate HSP diagnosis. Despite severity, a complete recovery is possible if diagnosed early and managed appropriately.

## Introduction

Henoch-Schönlein Purpura (HSP) is a small blood vessel vasculitis that primarily affects children and is uncommon among adult patients. The clinical tetrad of palpable purpura, arthralgia, abdominal pain, and renal abnormalities aids in the diagnosis. Hepatobiliary involvement in HSP cases is underreported, but the previous investigation suggested a transient change with complete recovery (1). At present, only two studies have studied the effect of obesity on HSP progression in children. Dundar *et al.* linked obesity with more severe cases of HSP due to a higher circulating proinflammatory cytokine (2). Studies in adult patients in this regard are still limited.

This case report aimed to highlight the unusual presentation of HSP in a 28-year-old obese male, with a detailed diagnosis and management plan. Most cases of HSP are self-limiting with an excellent prognosis and minimum clinical sequelae. Treatment is primarily supportive, including bed rest, adequate hydration, and monitoring renal and abdominal complications. HSP patients with hepatobiliary involvement given corticosteroids showed recovery within seven days (1). Nevertheless, HSP in adults requires routine follow-up due to a lower rate of complete recovery than in children, with a higher incidence of progression to chronic renal failure (3).

## Case Presentation

A 28-year-old obese male with no prior illness presented with a generalized skin eruption for three days. He initially experienced fever, cough, nasal congestion, and headache, followed by the appearance of a crop of asymptomatic purpura in the lower right limb. The patient was taken to the doctor and given antipyretic and antihistamine drugs. He denied any episodes of nausea, vomiting, or abdominal pain. A day before admission, the patient complained of a progressing skin rash in the lower and upper extremities, including the palms and soles. On admission, fever persisted despite antipyretic consumption, and a generalized, confluent rash was observed. The patient also complained of abdominal discomfort, arthralgia in both knees and ankles, and neck stiffness. He denied dysuria, blood-stained urine, and colicky abdominal pain. The patient had no prior medication use.

**Figure 1a-c:**
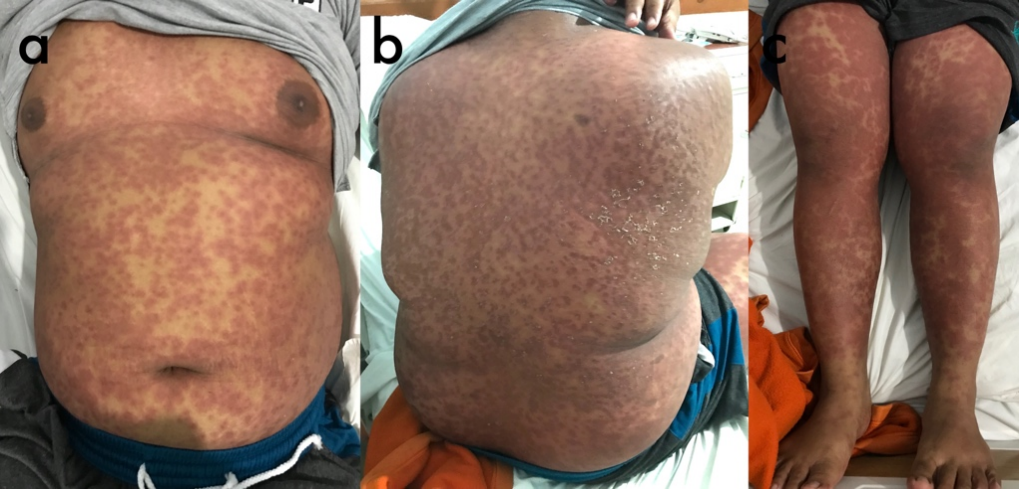
Cutaneous manifestation showed generalized, erythematous palpable purpura and some have coalesced to form ecchymoses.

Clinical examination revealed a fever of 39,6°C, and other vital signs were normal. Skin inspection revealed palpable, erythematous, purpuric, non-blanching eruptions with various sizes over the body (Figure 1). Cutaneous manifestation of the lower extremities showed the lesions had coalesced to form ecchymoses, with non-pitting pedal edema. Abdominal examination revealed no tenderness on palpation and the liver was not felt. There were no oral lesions, conjunctival injection, or any restriction of joint movements. Blood profile revealed leukocytosis (43.1 x 10^9^/L) with predominant granulocytes (39.2 x 10^9^/L), thrombocytosis (550 x 10^9^/L), and elevated transaminases (AST 125 U/L, ALT 131 U/L). Blood urea, creatinine levels, and urinalysis were normal.

Microscopic examination of the skin biopsy revealed the presence of a perivascular inflammatory infiltrate in the superficial dermal blood vessels, predominantly lymphocytes and a few neutrophils. A focal aggregate of inflammatory cells in the corneal skin layer was observed. Serum IgA level was not measured in this case.

The patient was given a high-calorie and high-protein diet, also rehydrated with normal saline. He was administered Cefuroxime dosed at 750mg twice daily for possible bacterial co-infection, along with intravenous pantoprazole and paracetamol for symptomatic treatment. He was initially started on intravenous methylprednisolone at 62.5 mg twice daily, but on the third day, it was escalated to 125mg twice daily due to increased purpuric rashes. On the fifth day, there was a resolution of symptoms with stable vital signs, and laboratory examinations returned to a normal range. The patient was then discharged and was switched to oral 16mg methylprednisolone twice daily and omeprazole tablets for stress ulcer prophylaxis for five days. The follow-up a week later showed no visible scars remained.

## Discussion

Henoch-Schönlein Purpura (HSP) is a small vessel vasculitis mediated by immunoglobulin A (IgA) deposition. Diagnosis of HSP requires the presence of palpable purpura in the lower limb, plus one of (1) Diffuse abdominal pain, (2) IgA deposits on biopsy, (3) Hematuria or proteinuria, (4) Acute arthralgias or arthritis. Demonstration of predominant IgA deposits in any biopsy becomes a requirement if a patient presents with atypical distribution of purpura. However, neither IgA staining nor serum level measurement was available in our center, though these data would have better supported our diagnosis. The triggers to HSP remain unclear, but prior infection has been suggested as a possibility, like in our case. Blood culture would have complemented antibiotic choice for our patient but was not feasible to perform in our center due to financial constraints.

To our best knowledge, no pediatric or adult HSP cases with a leukocyte count above 40,000 have been reported. We suggest the combination of infection, systemic vasculitis, and obesity be responsible for this occurrence. Obesity is associated with higher pro-inflammatory mediators and cytokines, such as tumor necrosis factor-α (TNF-α), interleukin-6 (IL-6), and adipokines. By understanding that HSP is a systemic vasculitis, IL-6 can induce thrombocytosis as an inflammatory reactive process (4). At present, only two studies have evaluated the effect of obesity on HSP in children in the literature. Dundar *et al.* suggested that increased adipose tissue contributed to HSP severity via higher levels of circulating mediators (2). Moreover, obese children with HSP had an increased risk of severe renal involvement by 3.1 fold. Literature showed the kidney is more commonly and severely involved in adults, with higher progression to renal failure. Our patient denied any history of flank pain or urinary complaints, denoted by normal renal function tests. High-dose corticosteroids and antibiotics administration reduced leukocyte and thrombocyte count, ruling out possible hematological disorders.

Hepatic involvement in HSP is scarcely reported. In China, a retrospective study observed mildly elevated liver enzyme levels in 9% cases and almost all recovered completely (1). Similarly, seven pediatric HSP cases in Italy had mildly elevated liver enzyme levels without hepatomegaly (5). Exercise was responsible for the altered liver profile in three children. The cause remained unknown in the remaining four patients but was speculated to be a result of infectious prodrome or from unreported self-administered drugs with potential liver toxicity. In our case, the patient denied medications or herbal consumption before onset. Before hospital admission, he has been prescribed dexamethasone, dexchlorpheniramine maleate, and paracetamol. These three active components that are all highly metabolized in the liver might have contributed to this phenomenon.

Although HSP is rare in adults, recognizing the clinical presentation will allow early diagnosis and prompt treatment. This case illustrates obesity and elevated transaminase levels in complicating HSP diagnosis, but the presence of palpable purpura with one other diagnostic sign should always raise suspicions for HSP. Despite its severe presentation, a complete recovery is possible with optimal management and close monitoring. This case report adds knowledge of the possible findings in adult HSP cases and serves as a driver for further research.
